# Gestational weight gain and pregnancy outcomes in Chinese women with type 2 diabetes mellitus: evidence from a tertiary hospital in Beijing

**DOI:** 10.3389/fendo.2024.1348382

**Published:** 2024-04-02

**Authors:** Xin Yan, Jianrui Jia, Wei Zheng, Xianxian Yuan, Jia Wang, Lirui Zhang, Guanghui Li

**Affiliations:** Division of Endocrinology and Metabolism, Department of Obstetrics, Beijing Obstetrics and Gynecology Hospital, Capital Medical University. Beijing Maternal and Child Health Care Hospital, Beijing, China

**Keywords:** diabetes mellitus, type 2, gestational weight gain, large for gestational age, pregnancy outcomes, diabetes

## Abstract

**Objective:**

To examine the effects of gestational weight gain on pregnancy outcomes and determine the optimal range of weight gain during pregnancy for Chinese women with type 2 diabetes mellitus.

**Methods:**

This retrospective cohort study included 691 Chinese women with type 2 diabetes mellitus from 2012 to 2020. The study utilized a statistical-based approach to determine the optimal range of gestational weight gain. Additionally, multivariate logistic regression analysis was conducted to assess the impact of gestational weight gain on pregnancy outcomes.

**Results:**

(1) In the obese subgroup, gestational weight gain below the recommendations was associated with decreased risks of large for gestational age (adjusted odds ratio [aOR] 0.19; 95% confidence interval [CI] 0.06-0.60) and macrosomia (aOR 0.18; 95% CI 0.05-0.69). In the normal weight subgroup, gestational weight gain below the recommendations of the Institute of Medicine was associated with decreased risks of preeclampsia (aOR 0.18; 95% CI 0.04-0.82) and neonatal hypoglycemia (aOR 0.38; 95% CI 0.15-0.97). (2) In the normal weight subgroup, gestational weight gain above the recommendations of the Institute of Medicine was associated with an increased risk of large for gestational age (aOR 4.56; 95% CI 1.54-13.46). In the obese subgroup, gestational weight gain above the recommendations was associated with an increased risk of preeclampsia (aOR 2.74; 95% CI 1.02, 7.38). (3) The optimal ranges of gestational weight gain, based on our study, were 9-16 kg for underweight women, 9.5-14 kg for normal weight women, 6.5-12 kg for overweight women, and 3-10 kg for obese women. (4) Using the optimal range of gestational weight gain identified in our study seemed to provide better prediction of adverse pregnancy outcomes.

**Conclusion:**

For Chinese women with type 2 diabetes, inappropriate gestational weight gain is associated with adverse pregnancy outcomes, and the optimal range of gestational weight gain may differ from the Institute of Medicine recommendations.

## Introduction

With the trend of delayed childbearing and the increasing prevalence of obesity, the worldwide prevalence of type 2 diabetes mellitus in pregnant women has been rapidly increasing (1). Between 1998 and 2013 in Scotland, as well as between 1998 and 2012 in Sweden, there was a significant increase in the number of pregnancies complicated by type 2 diabetes, with a respective rise of 90% and 111% (2, 3). A population-based cohort study conducted in China, which encompassed 6.4 million women aged 20-49 years old from 2010 to 2016, estimated the incidence of diabetes mellitus to be 1.18% (4). Diabetes mellitus was found to be associated with an increased risk of adverse maternal and neonatal outcomes, including hypertensive disorders of pregnancy, cesarean delivery, large for gestational age, macrosomia, congenital anomalies, stillbirth, and perinatal mortality (5–8). Furthermore, intrauterine exposure to diabetes increases the risk of developing obesity and diabetes in adulthood (9).

Previous research studies have reported that both gestational weight gain (GWG) and pre-pregnancy body mass index (BMI) are modifiable risk factors that can contribute to adverse pregnancy outcomes and have a direct influence on fetal development (8, 10, 11). Gestational weight gain has been found to be associated with a range of adverse pregnancy outcomes, including preterm delivery, cesarean section, hypertensive disorders of pregnancy, small for gestational age, large for gestational age, and macrosomia (11–13). According to the World Health Organization, global obesity rates nearly tripled from 1975 to 2016 and in 2016, over half of women aged 18 and older were overweight (40%) or obese (15%) (14). Pre-pregnancy obesity is strongly associated with adverse outcomes for both mothers and infants (15, 16). In 2009, the Institute of Medicine (IOM) updated GWG recommendations across BMI categories, of which gestational weight gain for underweight, normal weight, overweight, and obese women was 12.5–18, 11.5–16, 7–11.5, and 5–9 kg respectively (17). Population-based data from the Pregnancy Risk Assessment Monitoring System revealed that, according to the 2009 Institute of Medicine (IOM) recommendations, 20.9% of American women gained inadequate gestational weight, 32.0% gained adequate weight, and 47.2% gained excessive weight during pregnancy (18). However, it is important to note that the 2009 Institute of Medicine (IOM) recommendations have some limitations as they do not specifically address the optimal gestational weight gain (GWG) range for women with type 2 diabetes. While evidence suggests that the IOM guidelines are applicable to women with type 2 diabetes enrolled in California (19), it remains uncertain whether these guidelines are suitable for other populations, including Chinese women. Therefore, there is a need for information on the optimal GWG across maternal BMI categories for Chinese women with type 2 diabetes.

The objective of this study was to examine the effects of gestational weight gain on pregnancy outcomes and determine the optimal range of weight gain during pregnancy for Chinese women with type 2 diabetes mellitus.

## Materials and methods

### Study design and participants

This is a retrospective cohort study of women with type 2 diabetes mellitus who received perinatal care and gave birth at Beijing Obstetrics and Gynecology Hospital between January 1, 2012, and December 31, 2020. Participants were included in the study if they: (1) were aged between 18 and 45 years; (2) had a singleton pregnancy; (3) were diagnosed with type 2 diabetes mellitus (an established diagnosis of type 2 diabetes mellitus before pregnancy; or fasting glucose ≥ 7.0 mmol/L; or 2-hour plasma glucose ≥ 11.1 mmol/L during oral glucose tolerance test, or HbA1c ≥ 6.5%; or in a patient with classic symptoms of hyperglycemia or hyperglycemic crisis, a random plasma glucose ≥ 11.1 mmol/L) (20, 21); (4) delivered after 28 weeks of gestation. Participants were excluded if they had incomplete clinical data of gestational weight gain. A doctor thoroughly explained the informed consent form to the patient and answered all their questions and all participants signed informed consent documents prior to participation. This study was approved by the ethics committee of Beijing Obstetrics and Gynecology Hospital, Capital Medical University (2018-ky-009-01) and was carried out in accordance with the principles of the Declaration of Helsinki as revised in 2008. This manuscript was prepared according to STROBE statement (**Supplementary File 1**).

### Data collection

The electronic medical record system of Beijing Obstetrics and Gynecology Hospital was used to collect data, such as maternal demographic information (age, pre-pregnancy BMI, parity, and smoking status); medical history (chronic hypertension, thyroid disorders, and type 2 diabetes); complications and information during pregnancy (gestational weight gain, insulin therapy, hypertensive disorders of pregnancy, intrahepatic cholestasis of pregnancy and diabetic ketoacidosis); maternal and neonatal outcomes (placental abruption, delivery mode, postpartum hemorrhage, preterm birth, premature rupture of membrane, fetal distress, shoulder dystocia, neonatal sex, neonatal birthweight, neonatal intensive care unit admission, neonatal hypoglycemia, neonatal jaundice, and neonatal respiratory distress syndrome); and laboratory data.

### Definitions and protocols

All women followed up in our hospital were asked to provide their pre-pregnancy weight. Their heights were measured by a registered nurse at the first prenatal visit before 16 weeks of gestation. During pregnancy, all women were followed up every 4 weeks until 28 weeks of gestation, then every 2 weeks until 36 weeks of gestation, then weekly until delivery. At each prenatal visit, a registered nurse measured and recorded the patients’ weight and blood pressure. Women who were diagnosed with type 2 diabetes attended the hospital-based “one-day diabetes clinic,” which involved spending an entire day in the hospital for theory learning and practical training. Along with attending theoretical classes, they were also provided with a standard low glycemic index diet, participated in aerobics classes, and learned how to monitor their blood glucose levels on their own. Additionally, they were required to visit diabetes doctors every two weeks until delivery. Pre-pregnancy BMI, calculated as self-reported pre-pregnancy weight (in kilograms) divided by squared height (in meters), was categorized based on the World Health Organization recommendations as underweight (BMI, <18.5 kg/m^2^), normal weight (BMI, 18.5-24.9 kg/m^2^), overweight (BMI, 25-29.9 kg/m^2^), and obese (BMI, ≥30 kg/m^2^).

### Exposure

Overall GWG, calculated as weight before delivery minus the pre-pregnancy weight, was classified based on the Institute of Medicine (IOM) recommendations. Gestational weight gain below or above the recommendations was defined as inadequate or excessive weight gain.

### Outcomes

The primary outcome was large for gestational age (birthweight above the 90^th^ centile by gestational age and gender) (22). The secondary outcomes included hypertensive disorders (gestational hypertension and preeclampsia), delivery mode (cesarean section, assisted vaginal delivery, and vaginal delivery), preterm birth (<37 weeks), macrosomia (birthweight ≥ 4000g), small for gestational age (birthweight below the 10^th^ centile by gestational age and gender) (22), premature rupture of membranes, postpartum hemorrhage, neonatal hypoglycemia (<2.6mmol/L), neonatal jaundice (requiring phototherapy), neonatal respiratory distress syndrome, and neonatal intensive care unit admission.

### Determination of optimal GWG and GWG rate in the 2^nd^ and 3^rd^ trimesters

We used the statistical-based approach to determine the optimal range of GWG in women with type 2 diabetes mellitus.

The statistical-based approach was based on the distribution of GWG or GWG rate in the 2nd and 3rd trimesters in the “No complications subgroup”. The GWG rate in the 2nd and 3^rd^ trimesters was calculated as weight gain after 16 weeks of gestation divided by number of weeks from the 16 weeks of gestation to delivery. Women were divided into the “No complications subgroup” if they: (1) delivered at ≥37 weeks of gestation, (2) infant birth weight between 10^th^-90^th^ centile, (3) no maternal medical conditions (chronic hypertension and thyroid disorders), and no pregnancy complications (gestational hypertensive disorders, placental abruption, and intrahepatic cholestasis of pregnancy). Otherwise, women were defined as the “Complications subgroup”. In the “No complications subgroup”, the normal range of GWG or the GWG rate in the 2nd and 3^rd^ trimesters was defined as the interquartile range (IQR) of GWG or the GWG rate (25^th^ to 75^th^ centile of GWG or the GWG rate), which we considered reflecting the optimal range of GWG or GWG rate for women in our study. The distribution of gestational weight gain (GWG) and GWG rate were compared between two subgroups: the “No complications subgroup” and the “Complications subgroup”.

### Data analysis

All data were analyzed using the SPSS 26.0 software. Categorical variables of baseline characteristics and outcomes in the cohort stratified by pre-pregnancy BMI were expressed as numbers (percentages) and were compared using the Chi-square test or Fisher exact test. Continuous variables that did not conform to the normal distribution were expressed as median (P25, P75) and were compared using the Kruskal-Wallis test. Multivariable logistic regression analysis was used to assess the association of GWG below or above the optimal range with pregnancy outcomes, adjusting for the following confounders: maternal age, nulliparity, gestational age at delivery, chronic hypertension, type 2 diabetes diagnosed before or after pregnancy, HbA1c before 16 weeks of gestation, and the daily dose of insulin before delivery. Two-sided tests were employed for statistical evaluations, and P-values < 0.05 were considered statistically significant.

## Results

### Characteristics and outcomes of the study groups

**Figure 1:**
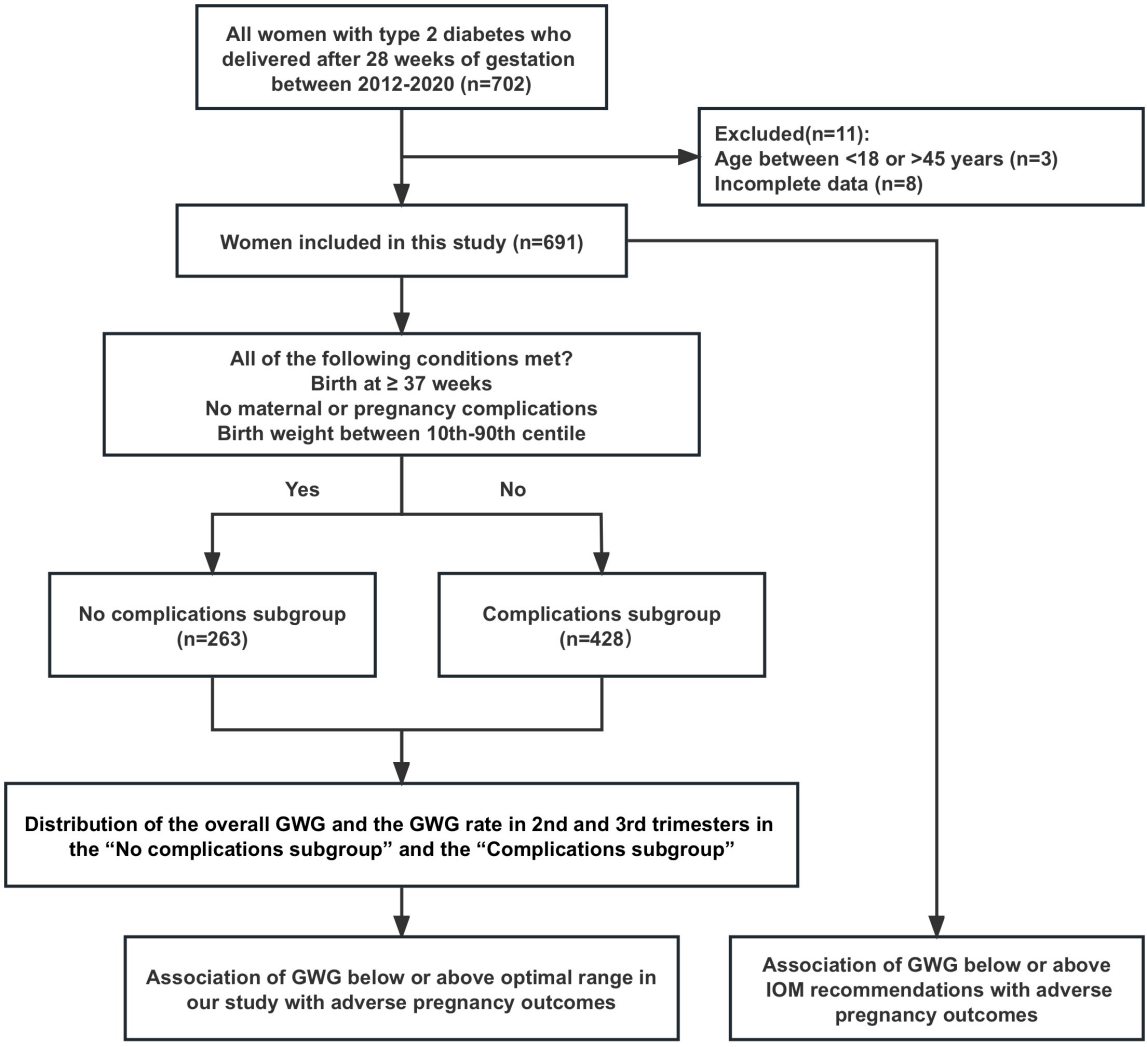
Flow chart for patient recruitment.

From 2012 to 2020, a total of 702 women with type 2 diabetes gave birth after 28 weeks of gestation at our hospital, and 11 patients who did not meet the included criteria were excluded (3 with age <18 or >45 and 8 with incomplete clinical data of gestational weight gain), then 691 women were finally included in this study. The 691 patients were classified into two subgroups: the “No complications subgroup” consisting of 263 patients, and the “Complications subgroup” consisting of 428 patients (**Figure 1**).

The baseline characteristics and outcomes of the overall cohort, stratified by pre-pregnancy BMI, were presented in **Table 1**. Among the 691 patients, 11 (1.59%) were underweight, 208 (30.10%) were of normal weight, 259 (37.48%) were overweight, and 213 (30.82%) were obese. There were 225(32.56%), 275(39.80%), and 191(27.64%) patients whose GWG was below, within, and above the 2009 IOM recommendations, respectively. Additionally, there were statistical differences in GWG among the pre-pregnancy BMI groups (P<0.001). The underweight group was the youngest, and the rate of advanced age was highest in the normal weight group (P=0.002 and P=0.007, respectively). The rates of type 2 diabetes diagnosed during pregnancy and cesarean section increased with higher pre-pregnancy BMI (both P<0.001), while the rate of vaginal delivery decreased (P=0.002). The rates of chronic hypertension and chronic hypertension with superimposed preeclampsia were lowest in the normal weight group and highest in the obese group (both P<0.001). The rate of small for gestational age was the highest in the underweight group and lowest in the overweight group (P=0.015). No significant statistical differences were observed in the other variables across the groups.

**Table 1:** Baseline characteristics and outcomes of the overall cohort stratified by pre-pregnancy BMI.

Characteristics	Overall (n=691)	Underweight (n=11)	Normal weight (n=208)	Overweight (n=259)	Obese (n=213)	P-value
Maternal age (years), Median (IQR)	34 (31, 37)	31 (28, 32)	35 (31.25, 37)	34 (31, 37)	33 (31, 36)	0.002^b^
>35years	259 (37.48)	1 (9.09)	88 (42.31)	106 (40.93)	64 (30.05)	0.007
Nulliparity	469 (67.87)	8 (72.73)	146 (70.19)	173 (66.80)	142 (66.67)	0.823
Smoking	1 (0.14)	0	0	0	1 (0.47)	0.718
Type 2 diabetes diagnosed during pregnancy	259 (37.48)	1 (9.09)	58 (27.88)	102 (39.38)	98 (46.01)	<0.001
Insulin therapy	602 (87.12)	9 (81.82)	181 (87.02)	223 (86.10)	189 (88.73)	0.799
Daily dose of insulin before delivery (IU), Median (IQR)	55 (26, 86)	24 (8, 48)	54 (23, 85)	58 (28, 90)	58 (26, 84)	0.076^b^
Chronic hypertension	106 (15.34)	1 (9.09)	10 (4.81)	34 (13.13)	61 (28.64)	<0.001
Thyroid disorders	78 (11.29)	1 (9.09)	20 (9.62)	25 (9.65)	32 (15.02)	0.231
Gestational weight gain						
Below IOM recommendations	225 (32.56)	7 (63.64)	93 (44.71)	67 (25.87)	58 (27.23)	<0.001
Within IOM recommendations	275 (39.80)	4 (36.36)	82 (39.42)	109 (42.08)	80 (37.56)	
Above IOM recommendations	191 (27.64)	0	33 (15.87)	83 (32.05)	75 (35.21)	
Pregnancy complications						
Gestational hypertension	70 (10.13)	0	20 (9.62)	30 (11.58)	20 (9.39)	0.566
Preeclampsia	79 (11.43)	1 (9.09)	16 (7.69)	32 (12.36)	30 (14.08)	0.201
Chronic hypertension with superimposed preeclampsia	43 (6.22)	1 (9.09)	4 (1.92)	12 (4.63)	26 (12.21)	<0.001^a^
Intrahepatic cholestasis of pregnancy	2 (0.29)	0	0	0	2 (0.94)	0.217^a^
Diabetic ketoacidosis	15 (2.17)	0	5 (2.40)	7 (2.70)	3 (1.41)	0.724^a^
Placental abruption	13 (1.88)	0	1 (0.50)	5 (1.90)	7 (3.30)	0.171^a^
Cesarean section	413 (59.77)	5 (45.45)	102 (49.04)	158 (61.00)	148 (69.48)	<0.001
Vaginal delivery	236 (34.15)	5 (45.45)	91 (43.75)	84 (32.43)	56 (26.29)	0.002
Assisted vaginal delivery	42 (6.08)	1 (9.09)	15 (7.21)	17 (6.56)	9 (4.23)	0.422^a^
Postpartum hemorrhage	102 (14.76)	0	26 (12.5)	44 (16.99)	32 (15.02)	0.286
Preterm birth	85 (12.30)	1 (9.09)	17 (8.17)	33 (12.74)	34 (15.96)	0.108
Premature rupture of membrane	172 (24.89)	5 (45.50)	57 (27.40)	65 (25.10)	45 (21.10)	0.186
Fetal distress	124 (17.95)	3 (27.27)	30 (14.42)	56 (21.62)	35 (16.43)	0.164
Gestational age at delivery (weeks), Median (IQR)	38 (38, 39)	38 (37, 38)	38 (38, 39)	38 (38, 39)	38 (37, 39)	0.070^b^
Shoulder dystocia	16 (2.32)	1 (9.09)	6 (2.88)	6 (2.32)	3 (1.41)	0.240^a^
Neonatal outcomes						
Sex/male	360 (52.10)	8 (72.73)	110 (52.88)	133 (51.35)	109 (51.17)	0.560
Sex/female	331 (47.90)	3 (27.27)	98 (47.12)	126 (48.65)	104 (48.83)	
Small for gestational age	23 (3.33)	3 (27.27)	7 (3.37)	7 (2.70)	6 (2.82)	0.015^a^
Large for gestational age	143 (20.69)	1 (9.09)	36 (17.31)	53 (20.46)	54 (25.35)	0.162
Macrosomia	112 (16.21)	1 (9.09)	26 (12.50)	44 (16.99)	41 (19.25)	0.253
NICU admission	197 (28.51)	4 (36.36)	45 (21.63)	84 (32.43)	64 (30.05)	0.061
Neonatal hypoglycemia	107 (15.48)	0	32 (15.38)	38 (14.67)	37 (17.37)	0.436
Neonatal jaundice	86 (12.45)	0	21 (10.10)	42 (16.20)	23 (10.80)	0.089
Neonatal respiratory distress syndrome	62 (8.97)	2 (18.18)	16 (7.69)	25 (9.65)	19 (8.92)	0.518^a^

Values are expressed as number (percentage), unless indicated otherwise.

IQR, Inter Quartile Range; IOM, Institute of Medicine; NICU, neonatal intensive care unit.

^a^Fisher exact test; ^b^ Kruskal–Wallis test.

### Association of GWG below or above IOM recommendations with adverse pregnancy outcomes

The underweight group was not analyzed because of the small sample size. In the obese subgroup, GWG below the IOM recommendations was associated with lower risks of large for gestational age (OR 0.19; 95%CI 0.06-0.60) and macrosomia (OR 0.18; 95%CI 0.05-0.69) (**Table 2**). GWG below the IOM recommendations in the normal weight subgroup was associated with lower risks of preeclampsia (OR 0.18; 95%CI 0.04-0.82) and neonatal hypoglycemia (OR 0.38; 95%CI 0.15-0.97). In addition, GWG above the IOM recommendations in the normal weight subgroup was associated with higher risk of large for gestational age (OR 4.56; 95%CI 1.54-13.46) (**Table 2**).

**Table 2:** Association of GWG below and above the 2009 IOM recommendations with adverse pregnancy outcomes.

BMI group	Outcomes	Rate of outcome				Risk of outcome	
		GWG below IOM	GWG within IOM	GWG above IOM	P-value	GWG below VS within IOM	GWG above VS within IOM
						aOR (95%CI)	aOR (95%CI)
Normal weight	Cesarean delivery	41 (44.09)	40 (48.78)	21 (63.64)	0.155	0.77 (0.41, 1.46)	1.57 (0.65, 3.78)
	Assisted vaginal delivery	7 (7.53)	6 (7.32)	2 (6.06)	0.961	1.34 (0.39, 4.68)	0.78 (0.13, 4.67)
	Vaginal delivery	45 (48.39)	36 (43.90)	10 (30.30)	0.198	1.19 (0.63, 2.23)	0.66 (0.26, 1.64)
	Gestational hypertension	6 (6.45)	9 (10.98)	5 (15.15)	0.300	0.57 (0.18, 1.85)	1.19 (0.34, 4.24)
	Preeclampsia	4 (4.30)	9 (10.98)	3 (9.09)	0.241	0.18 (0.04, 0.82)^b^	0.53 (0.12, 2.34)
	PROM	29 (31.18)	21 (25.61)	7 (21.21)	0.488	1.22 (0.61, 2.42)	0.89 (0.32, 2.42)
	Preterm birth	9 (9.68)	7 (8.54)	1 (3.03)	0.482	/	/
	Postpartum hemorrhage	10 (10.75)	12 (14.63)	4 (12.12)	0.739	0.74 (0.29, 1.90)	0.88 (0.25, 3.12)
	Macrosomia	6 (6.45)	12 (14.63)	8 (6.45)	0.022^b^	0.38 (0.12, 1.21)	3.09 (0.91, 10.44)
	Small for gestational age	3 (3.23)	3 (3.66)	1 (3.03)	1^a^	0.74 (0.11, 4.84)	0.96 (0.06, 14.49)
	Large for gestational age	9 (9.68)	15 (18.29)	12 (36.36)	0.002^b^	0.38 (0.14, 1.03)	4.56 (1.54, 13.46)^b^
	Neonatal hypoglycemia	8 (20.73)	17 (20.73)	7 (21.21)	0.051	0.38 (0.15, 0.97)^b^	0.89 (0.30, 2.63)
	Neonatal jaundice	8 (8.60)	9 (10.98)	4 (12.12)	0.799	0.77 (0.25, 2.33)	1.09 (0.28, 4.30)
	NRDS	8 (8.60)	5 (6.10)	3 (8.60)	0.782	1.24 (0.30, 5.20)	2.54 (0.44, 14.71)
	NICU admission	18 (19.35)	19 (23.17)	8 (24.24)	0.767	0.63 (0.27, 1.51)	0.97 (0.33, 2.80)
Overweight	Cesarean delivery	36 (53.73)	61 (55.96)	61 (73.49)	0.017^b^	0.87 (0.45, 1.67)	1.71 (0.88, 3.33)
	Assisted vaginal delivery	3 (4.48)	8 (7.34)	6 (7.23)	0.725	0.49 (0.11, 2.15)	1.07 (0.31, 3.71)
	Vaginal delivery	28 (41.79)	40 (36.70)	16 (19.28)	0.006^b^	1.34 (0.69, 2.59)	0.54 (0.26, 1.11)
	Gestational hypertension	4 (5.97)	12 (11.01)	14 (16.87)	0.113	0.42 (0.12, 1.40)	2.36 (0.92, 6.05)
	Preeclampsia	9 (13.43)	11 (10.09)	12 (13.43)	0.629	1.30 (0.48, 3.53)	1.34 (0.51, 3.57)
	PROM	19 (25.69)	28 (25.69)	18 (21.69)	0.634	1.02 (0.50, 2.07)	0.73 (0.35, 1.52)
	Preterm birth	10 (11.93)	13 (11.93)	10 (12.05)	0.823	/	/
	Postpartum hemorrhage	6 (8.96)	23 (21.10)	15 (18.07)	0.108	0.38 (0.14, 1.02)	0.66 (0.29, 1.47)
	Macrosomia	10 (14.93)	17 (15.60)	17 (14.93)	0.586	0.90 (0.36, 2.26)	1.78 (0.76, 4.19)
	Small for gestational age	4 (5.97)	3 (2.75)	0	0.057^a^	3.07 (0.53, 17.64)	/
	Large for gestational age	11 (16.42)	20 (18.35)	22 (26.51)	0.242	0.83 (0.35 1.97)	1.91 (0.86, 4.24)
	Neonatal hypoglycemia	7 (10.45)	16 (14.68)	15 (18.07)	0.423	0.69 (0.26, 1.82)	1.10 (0.47, 2.58)
	Neonatal jaundice	10 (14.93)	18 (16.51)	14 (16.87)	0.944	0.88 (0.37, 2.13)	0.90 (0.39, 2.07)
	NRDS	7 (10.45)	10 (9.17)	8 (9.64)	0.962	0.87 (0.25, 3.02)	0.70 (0.20, 2.38)
	NICU admission	24 (35.82)	31 (28.44)	29 (34.94)	0.501	1.17 (0.57, 2.40)	1.25 (0.60, 2.60)
Obese	Cesarean delivery	34 (58.62)	54 (67.50)	60 (80.00)	0.026^b^	0.57 (0.27, 1.22)	1.56 (0.72, 3.37)
	Assisted vaginal delivery	4 (6.90)	4 (5.00)	1 (1.33)	0.285^a^	1.88 (0.37, 9.49)	0.37 (0.04, 3.74)
	Vaginal delivery	20 (34.48)	22 (27.50)	14 (18.67)	0.115	1.57 (0.72, 3.41)	0.73 (0.33, 1.61)
	Gestational hypertension	3 (5.17)	9 (11.25)	8 (10.67)	0.431	0.45 (0.11, 1.87)	1.17 (0.39, 3.46)
	Preeclampsia	5 (8.62)	9 (11.25)	16 (21.33)	0.074	0.73 (0.21, 2.58)	2.74 (1.02, 7.38)^b^
	PROM	14 (24.14)	16 (20.00)	15 (20.00)	0.805	1.60 (0.68, 3.78)	0.97 (0.43, 2.20)
	Preterm birth	9 (15.52)	14 (17.50)	11 (14.67)	0.885	/	/
	Postpartum hemorrhage	10 (17.24)	11 (13.75)	11 (14.67)	0.847	1.12 (0.43, 2.95)	1.15 (0.45, 2.92)
	Macrosomia	3 (5.17)	16 (20.00)	22 (29.33)	0.002^b^	0.18 (0.05, 0.69)^b^	1.76 (0.77, 4.02)
	Small for gestational age	3 (5.17)	0	3 (4.00)	0.111^a^	/	/
	Large for gestational age	4 (6.90)	21 (26.25)	29 (38.67)	<0.001^b^	0.19 (0.06, 0.60)^b^	1.70 (0.82, 3.56)
	Neonatal hypoglycemia	9 (15.52)	14 (17.50)	14 (18.67)	0.892	0.80 (0.30, 2.11)	0.87 (0.37, 2.05)
	Neonatal jaundice	7 (12.07)	12 (15.00)	4 (5.33)	0.143	0.76 (0.26, 2.22)	0.35 (0.10, 1.19)
	NRDS	6 (10.34)	7 (8.75)	6 (8.00)	0.893	1.55 (0.40, 6.02)	1.15 (0.30, 4.46)
	NICU admission	19 (32.76)	21 (26.25)	24 (32.00)	0.642	1.47 (0.62, 3.48)	1.11 (0.50, 2.44)

Values are expressed as number (percentage). GWG, gestational weight gain; IOM, Institute of Medicine; BMI, body mass index; aOR, adjusted odds ratio; CI, confidence interval; PROM, premature rupture of membranes; NRDS, neonatal respiratory distress syndrome; NICU, neonatal intensive care unit; ^a^Fisher exact test; ^b^Significant associations.

### Optimal overall GWG and GWG rate in the 2^nd^ and 3^rd^ trimesters: a statistical-based approach

**Figure 2:**
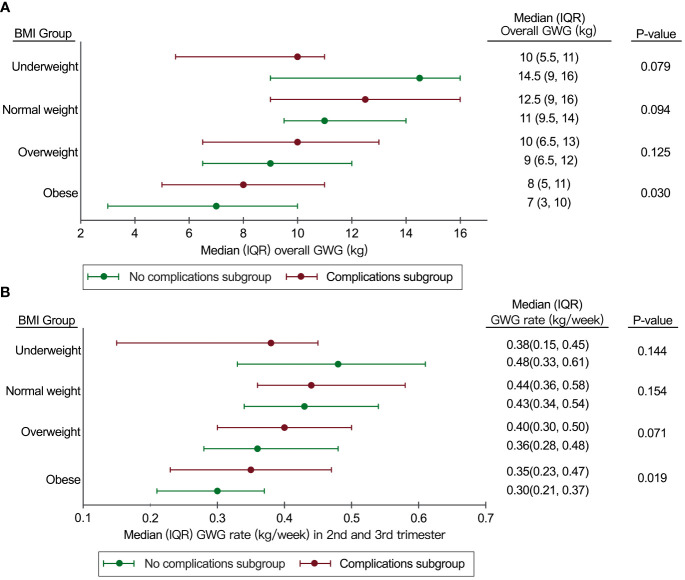
Distribution of overall GWG and GWG rate in 2nd and 3rd trimesters in the “No complications subgroup” and the “Complications subgroup”. **(A)** Distribution of overall GWG in the “No complications subgroup” and the “Complications subgroup”; **(B)** Distribution of GWG rate in the 2nd and 3rd trimesters in the “No complications subgroup” and the “Complications subgroup”. BMI, body mass index; GWG, gestational weight gain; IQR, interquartile range.

In order to determine the optimal range of GWG and GWG rates in the 2nd and 3rd trimesters for our study, we analyzed the distribution of overall GWG and GWG rates specifically in the “No complications subgroup” and the “Complications subgroup” (**Figure 2**).

The IQR of GWG for the overweight group in this study was similar to the IOM recommendations (6.5-12kg in our study vs 7-11.5kg in the IOM recommendations), but the IQR of GWG for the underweight group (9-16kg in our study vs 12.5-18kg in the IOM recommendations) and the normal weight group (9.5-14kg in our study vs 11.5-16kg in the IOM recommendations) in our study was lower than the IOM recommendations (**Figure 2A**).

Compared to the IOM recommendations, the IQR of GWG rate in the 2^nd^ and 3^rd^ trimesters in the normal weight group was similar (0.34-0.54kg/week in our study vs 0.35-0.50kg/week in the IOM recommendations). However, the IQR of GWG rate in 2^nd^ and 3^rd^ trimesters for the overweight group (0.28-0.48kg/week in our study vs 0.23-0.33kg/week in the IOM recommendations) and the obese group (0.21-0.37 kg/week in our study vs 0.17-0.27 kg/week in the IOM recommendations) in our study was higher than the IOM recommendations (**Figure 2B**).

### Association of GWG below or above recommendations in our study with adverse pregnancy outcomes

The underweight group was not analyzed because of the small sample size. There were 48.91% of women who gained inappropriate GWG compared to the optimal range of GWG in our study (22.00% below the optimal range and 26.91% above the optimal range, respectively).

In the overweight subgroup, GWG above the optimal range of GWG in our study was associated with increased risks of large for gestational age (OR 2.38; 95%CI 1.06-5.36), macrosomia (OR 2.48; 95%CI 1.03-5.96), and gestational hypertension (OR 2.73; 95%CI 1.07, 6.98), which was not observed when IOM recommendations were used to define excessive GWG. In the obese subgroup, GWG above the optimal range of GWG in our study was associated with increased risks of large for gestational age (OR 3.27; 95%CI 1.55-6.89), macrosomia (OR 3.30; 95%CI 1.45-7.55), cesarean section (OR 3.31; 95%CI 1.40-7.84), and preeclampsia (OR 4.12; 95%CI 1.58-10.75), but a decreased rate of vaginal delivery (OR 0.33, 95%CI 0.14-0.82), which was not observed when IOM recommendations were used to define excessive GWG (**Table 3**). In the normal weight subgroup, GWG above the optimal range of GWG in our study was associated with increased risks of macrosomia (OR 4.74; 95%CI 1.48-15.17) and neonatal respiratory distress syndrome (OR 7.18; 95%CI 1.24-41.61), which was not observed when IOM recommendations were used to define excessive GWG.

**Table 3:** Association of GWG below and above the optimal range in the current study with adverse pregnancy outcomes.

BMI group	Outcomes	Rate of outcome				Risk of outcome	
		GWG below the optimal range	GWG within the optimal range	GWG above the optimal range	P-value	GWG below VS within the optimal range	GWG above VS within the optimal range
						aOR (95%CI)	aOR (95%CI)
Normal weight	Cesarean delivery	21 (41.18)	48 (48.98)	33 (55.93)	0.304	0.68 (0.33, 1.41)	1.26 (0.62, 2.56)
	Assisted vaginal delivery	4 (7.84)	5 (5.10)	6 (10.17)	0.448^a^	1.42 (0.32, 6.24)	1.45 (0.38, 5.62)
	Vaginal delivery	26 (50.98)	45 (45.92)	20 (33.9)	0.166	1.32 (0.64, 2.72)	0.69 (0.33, 1.44)
	Gestational hypertension	3 (5.88)	8 (8.16)	9 (15.25)	0.200	0.55 (0.12, 2.51)	1.64 (0.54, 4.97)
	Preeclampsia	3 (5.88)	8 (8.16)	5 (8.47)	0.892^a^	0.31 (0.05 1.91)	0.88 (0.25, 3.10)
	PROM	14 (27.45)	31 (31.63)	12 (20.34)	0.307	0.69 (0.31, 1.52)	0.61 (0.27, 1.37)
	Preterm birth	7 (13.73)	8 (8.16)	2 (3.39)	0.146^a^	/	/
	Postpartum hemorrhage	6 (14.29)	14 (14.29)	6 (10.17)	0.739	0.76 (0.26, 2.27)	0.57 (0.19, 1.70)
	Macrosomia	1 (1.96)	11 (11.22)	14 (23.73)	0.002^b^	0.12 (0.01, 1.10)	4.74 (1.48, 15.17)^b^
	Small for gestational age	3 (5.88)	2 (2.04)	2 (3.39)	0.417^a^	3.55 (0.43, 29.01)	2.05 (0.19, 22.13)
	Large for gestational age	3 (5.88)	15 (15.31)	18 (30.51)	0.002^b^	0.25 (0.06, 1.00)	4.30 (1.61, 11.48)^b^
	Neonatal hypoglycemia	6 (11.76)	16 (16.33)	10 (16.95)	0.708	0.71 (0.25, 2.07)	0.74 (0.28, 1.96)
	Neonatal jaundice	5 (7.10)	7 (7.10)	9 (15.30)	0.262	1.33 (0.36, 5.02)	2.35 (0.73, 7.57)
	NRDS	6 (3.06)	3 (3.06)	7 (11.86)	0.040^ab^	1.86 (0.31, 11.17)	7.18 (1.24, 41.61)^b^
	NICU admission	11 (21.57)	18 (18.37)	16 (21.57)	0.435	0.62 (0.21, 1.86)	1.66 (0.69, 4.00)
Overweight	Cesarean delivery	33 (53.23)	74 (57.81)	51 (53.23)	0.031^b^	0.80 (0.42, 1.54)	1.55 (0.77, 3.10)
	Assisted vaginal delivery	3 (4.84)	10 (7.81)	4 (5.80)	0.804^a^	0.50 (0.12, 2.14)	0.74 (0.20, 2.78)
	Vaginal delivery	26 (41.94)	44 (34.38)	14 (20.29)	0.025^b^	1.47 (0.76, 2.85)	0.68 (0.32, 1.43)
	Gestational hypertension	3 (4.84)	14 (10.94)	13 (18.84)	0.042^b^	0.32 (0.09, 1.22)	2.73 (1.07, 6.98)^b^
	Preeclampsia	8 (12.9)	14 (10.94)	10 (14.49)	0.761	1.13 (0.42, 3.06)	1.20 (0.46, 3.14)
	PROM	17 (27.42)	35 (27.34)	13 (18.84)	0.376	0.90 (0.45, 1.82)	0.55 (0.25, 1.19)
	Preterm birth	10 (16.13)	15 (11.72)	8 (11.59)	0.656	/	/
	Postpartum hemorrhage	6 (9.68)	27 (21.09)	11 (15.94)	0.140	0.41 (0.16, 1.09)	0.51 (0.22, 1.22)
	Macrosomia	10 (16.13)	18 (14.06)	16 (23.19)	0.261	1.09 (0.44, 2.72)	2.48 (1.03, 5.96)^b^
	Small for gestational age	3 (4.84)	4 (3.13)	0	0.214	1.98 (0.37, 10.68)	/
	Large for gestational age	11 (17.74)	22 (17.19)	20 (28.99)	0.122	0.98 (0.41, 2.30)	2.38 (1.06, 5.36)^b^
	Neonatal hypoglycemia	7 (11.29)	20 (15.63)	11 (15.94)	0.688	0.70 (0.27, 1.82)	0.81 (0.33, 1.97)
	Neonatal jaundice	10 (16.13)	19 (14.84)	13 (18.84)	0.768	1.10 (0.46, 2.63)	1.21 (0.52, 2.83)
	NRDS	7 (11.29)	11 (8.59)	7 (10.14)	0.829	0.97 (0.29, 3.33)	0.736 (0.21, 2.62)
	NICU admission	23 (37.10)	39 (30.47)	22 (31.88)	0.654	1.12 (0.55, 2.28)	0.88 (0.42, 1.86)
Obese	Cesarean delivery	23 (65.71)	76 (62.81)	49 (85.96)	0.006^b^	1.06 (0.45, 2.46)	3.31 (1.40, 7.84)^b^
	Assisted vaginal delivery	2 (5.71)	6 (4.96)	1 (1.75)	0.640^a^	1.98 (0.30, 13.17)	0.45 (0.05, 4.16)
	Vaginal delivery	10 (28.57)	39 (32.23)	7 (12.28)	0.018^b^	0.85 (0.36, 2.03)	0.33 (0.14, 0.82)^b^
	Gestational hypertension	3 (8.57)	11 (9.09)	6 (10.53)	0.939	0.99 (0.24, 4.05)	1.41 (0.47, 4.29)
	Preeclampsia	2 (5.71)	14 (11.57)	14 (24.56)	0.020^b^	0.50 (0.10, 2.56)	4.12 (1.58, 10.75)^b^
	PROM	7 (20.00)	26 (21.49)	12 (21.05)	0.982	1.12 (0.42, 2.95)	0.94 (0.42, 2.09)
	Preterm birth	7 (20.00)	19 (15.70)	8 (14.04)	0.745	/	/
	Postpartum hemorrhage	5 (14.29)	18 (14.88)	9 (15.79)	0.979	0.78 (0.26, 2.39)	1.10 (0.45, 2.71)
	Macrosomia	3 (8.57)	19 (15.70)	19 (33.33)	0.004^b^	0.47 (0.12, 1.80)	3.30 (1.45, 7.55)^b^
	Small for gestational age	2 (5.71)	2 (1.65)	2 (3.51)	0.251^a^	3.71 (0.44, 31.08)	2.23 (0.27, 18.45)
	Large for gestational age	3 (8.57)	26 (21.49)	25 (43.86)	<0.001^b^	0.32 (0.09, 1.17)	3.27 (1.55, 6.89)^b^
	Neonatal hypoglycemia	8 (22.86)	19 (15.70)	10 (17.54)	0.616	1.83 (0.69, 4.86)	0.97 (0.40, 2.33)
	Neonatal jaundice	3 (8.57)	18 (14.88)	2 (3.51)	0.067	0.54 (0.14, 2.07)	0.22 (0.05, 1.02)
	NRDS	5 (14.29)	8 (6.61)	6 (10.53)	0.330	3.66 (0.88, 15.27)	2.57 (0.65, 10.19)
	NICU admission	12 (34.29)	32 (26.45)	20 (35.09)	0.420	1.70 (0.67, 4.30)	1.37 (0.64, 2.92)

Values are expressed as number (percentage). GWG, gestational weight gain; BMI, body mass index; aOR, adjusted odds ratio; CI, confidence interval; PROM, premature rupture of membranes; NRDS, neonatal respiratory distress syndrome; NICU, neonatal intensive care unit; ^a^Fisher exact test; ^b^Significant associations.

## Discussion

In our study, more than half of the women with type 2 diabetes experienced inappropriate GWG compared to the 2009 IOM recommendations, which was consistent with previous research (23). We found that GWG below the IOM recommendations in women with type 2 diabetes was associated with lower risks of large for gestational age, macrosomia, and preeclampsia, which was consistent with prior studies (24, 25). Previous studies found that GWG above IOM recommendations increased the risk of neonatal hypoglycemia (26). However, we found GWG below the IOM recommendations was associated with lower risk of neonatal hypoglycemia. In the general population, GWG above IOM recommendations decreased the risk of small for gestational age (12). Nevertheless, in our study, excessive GWG in women with type 2 diabetes was not a protective factor for small for gestational age, which was consistent with previous studies (19, 24, 27).

It is worth noting that the optimal range of GWG for Chinese women with type 2 diabetes in our study is different from the IOM recommendations. In September 2022, the Chinese Center for Disease Control and Prevention released the standard of recommendation for weight gain during pregnancy based on data including more than 100 000 singleton pregnant Chinese women, in which the GWG recommendations for underweight (BMI, <18.5 kg/m (2)), normal weight (BMI, 18.5-24 kg/m^2^), overweight (BMI, 24-28 kg/m^2^) and obese women (BMI, ≥28 kg/m^2^) were 11-16kg, 8-14kg, 7-11 kg, and 5-9kg, respectively (28). The Chinese recommendations for underweight women and normal-weight women are lower than the IOM recommendations, which is roughly consistent with the results of this study. And a systematic review and meta-analysis evaluating GWG across continents and ethnicity indicated that IOM guidelines might not be suitable for Asian women (29). This partly explains why using the optimal range of GWG from our study to define excessive GWG seems to predict adverse pregnancy outcomes in Chinese women with type 2 diabetes better than the IOM recommendations.

GWG is a significant and modifiable risk factor for pregnancy outcomes in pregnant women with type 2 diabetes. The physiologic mechanisms that inappropriate gestational weight gain in women with type 2 diabetes might cause adverse pregnancy outcomes were under research, and there were plausible mechanisms, such as adverse effects of hyperglycemia, insulin resistance, and high pre-pregnancy BMI (30–33). However, the specific regulatory mechanisms between GWG in women with type 2 diabetes and adverse pregnancy outcomes need to be further studied. We determined an optimal range of GWG for women with type 2 diabetes in our study, however, whether this optimal range is suitable for Chinese women with type 2 diabetes still needs to be further confirmed by higher-quality, large-sample, and multi-center research. Determining the optimal GWG for women with type 2 diabetes is essential for avoiding adverse pregnancy outcomes (34).

### Strengths and limitations

To our knowledge, this is the first research to evaluate the overall GWG and the GWG rate in the 2^nd^ and 3^rd^ trimesters in women with type 2 diabetes, and this study is also the first research to investigate the relationship between GWG according to the IOM recommendations and pregnancy outcomes in Chinese women with type 2 diabetes. We recognize that our study did not include variables such as diet and physical exercise, which represents a significant limitation in assessing the impacts of GWG in women with type 2 diabetes. Diet and physical exercise are known to critically influence weight gain and glycemic control during pregnancy. These factors could significantly modulate the relationship between GWG and pregnancy outcomes in women with type 2 diabetes. Therefore, the effects of diet and physical exercise on GWG during pregnancy in this specific population, as well as their interactions, warrant further in-depth research and exploration. Future studies should prioritize these variables to comprehensively understand the complex mechanisms by which GWG affects pregnancy outcomes. Additionally, while we have utilized a data span of 8 years, only 691 individuals met the criteria for our study. The limited sample size restricts the generalizability of our research findings. It is necessary to conduct multicenter, large-sample studies to validate the findings regarding gestational weight gain in this specific population.

## Conclusion

Our study suggests that a large proportion of Chinese women with type 2 diabetes experienced inappropriate GWG and it is associated with adverse pregnancy outcomes. For Chinese women with type 2 diabetes, the optimal range of GWG might be different from IOM recommendations. Further studies are needed to validate the findings regarding gestational weight gain in this specific population.

## Data availability statement

The original contributions presented in the study are included in the article/**Supplementary Materials**, further inquiries can be directed to the corresponding author/s.

## Ethics statement

The studies involving humans were approved by the ethics committee of Beijing Obstetrics and Gynecology Hospital, Capital Medical University (2018-ky-009-01). The studies were conducted in accordance with the local legislation and institutional requirements. The participants provided their written informed consent to participate in this study.

## Author contributions

XY: Conceptualization, Formal analysis, Investigation, Software, Writing – original draft. JJ: Conceptualization, Formal analysis, Investigation, Software, Writing – original draft. WZ: Methodology, Writing – review & editing. XXY: Methodology, Writing – review & editing. JW: Data curation, Writing – review & editing. LZ: Data curation, Writing – review & editing. GL: Funding acquisition, Investigation, Project administration, Supervision, Writing – review & editing.

## Supplementary material

Supplementary File 1 STROBE Statement—checklist of items that should be included in reports of observational studies.
